# Cavitating pulmonary lesion secondary to mycobacterium szulgai infection—case report of a rare pathogenic organism

**DOI:** 10.1093/omcr/omae072

**Published:** 2024-07-13

**Authors:** Aroon Sohail, James Hare, Abdalla Ibrahim

**Affiliations:** Lancaster Medical School, Lancaster University, Lancaster LA1 4YW, United Kingdom; Department of Medicine, Warrington and Halton Hospitals NHS Trust, Lovely Lane, Warrington WA5 1QG, United Kingdom; Department of Radiology, Warrington and Halton Hospitals NHS Trust, Lovely Lane, Warrington, WA5 1QG, United Kingdom; Department of Respiratory Medicine, Warrington and Halton Hospitals NHS Trust, Lovely Lane, Warrington, WA5 1QG, United Kingdom

**Keywords:** respiratory medicine, diagnostic radiology, atypical infection

## Abstract

*Mycobacterium szulgai* (MS) is a species of non-tuberculous mycobacterium (NTM), which very rarely is identified as the causative pathogen of pulmonary infections. Due to its rarity, there are limitations in the existing literature regarding the diagnosis, investigation and treatment of MS pulmonary infection. Our case report provides further information regarding the clinical, microbiological and radiological findings associated with MS pulmonary infection with suggestions provided on its long term management.

## Introduction

Non-tuberculous mycobacteria (NTM) are species of mycobacterium which are separate to the *Mycobacterium tuberculosis* complex and *Mycobacterium leprae* [1]. Although uncommon, NTM can cause a variety of human infections, of which pulmonary infections are amongst the most frequent [2]. NTM pulmonary infections can lead to progressive inflammatory lung damage, a condition named NTM-pulmonary disease (NTM-PD). The rate of NTM pulmonary infections has increased in the United Kingdom (UK) from 4.0/100 000 in 2007 to 6.1/100 000 in 2012 [3]. Amongst the more than 170 species of NTM identified, *Mycobacterium szulgai* (MS) as the causative pathogen of NTM-PD remains exceedingly rare, leading to limitations in understanding the clinical presentation, radiological findings and response to treatment in MS NTM-PD. Herein, we present a case of an elderly man with NTM-PD secondary to MS infection.

## Case report

A 74 year old man presented to the local accident and emergency department with a 6 week history of progressive dyspnoea and productive cough of black and yellow sputum. He described progressive fatigue, cold sweats in the night and a reduction in appetite associated with weight loss of 6.5 kilograms over a year. He had a 60 pack-year history of smoking. Comorbidities included; chronic obstructive pulmonary disease (COPD), ischaemic dilated cardiomyopathy, ischaemic heart disease and atrial fibrillation.

His observation parameters were unremarkable, with a respiratory rate of 19 and oxygen saturations on room air of 95%. He was haemodynamically stable and apyrexic. On auscultation, there was global expiratory wheeze in both lungs with crepitations audible in the left lung apex. His white blood cell count and neutrophil count were normal, with a mildly raised C-reactive protein of 37. His arterial blood gas on room air showed no significant abnormality.

**Figure 1:**
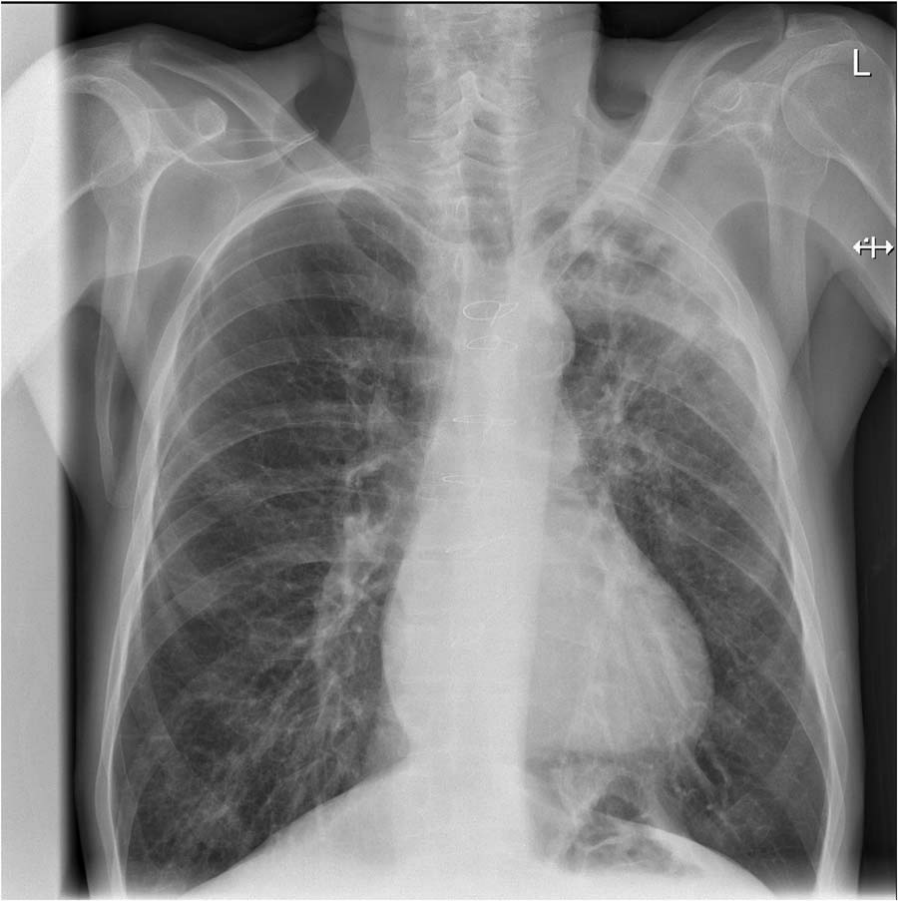
Posterior–Anterior chest radiograph showing a cavity at the left apex with surrounding consolidation. This was not present on the prior radiograph obtained nine months earlier.

**Figure 2:**
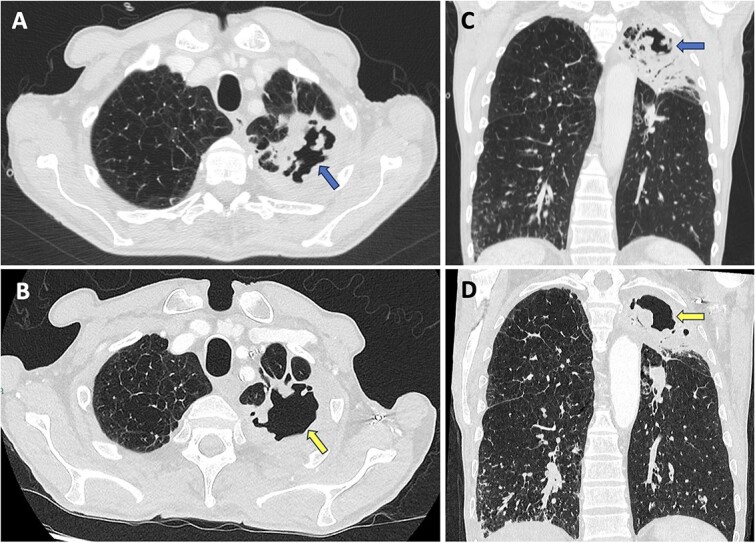
Images A and C are axial and coronal CT slices showing the cavity in the left upper lobe with surrounding consolidation (blue arrows). Images B and D are axial and coronal CT slices acquired 11 weeks later showing an increase in size of the cavity, with persistent surrounding consolidation (yellow arrows).

A chest radiograph was performed, which demonstrated increased airspace change within the left upper zone (Fig. 1). A subsequent computed tomography of the thorax (CT-T) with contrast demonstrated significant consolidation in the left upper lobe with air bronchograms and a large area of central necrotic change with cavitation measuring 3.3 cm × 5 cm × 4.6 cm (Fig. 2). Empirical antibiotics were started to cover for an atypical chest infection. Sputum samples were sent, two of which were positive for *Mycobacterium Sp*. with a third identifying *M. szulgai*. The time from sputum sampling to species identification was 8 weeks. Aspergillus IgG, serum galactomannan, beta-D glucan and acid fast bacillus tests were all negative, indicating no active aspergillus, fungal or *M. tuberculosis* pulmonary infection. The case was discussed in the regional Tuberculosis multi-disciplinary team (TB-MDT) meeting, with the outcome of starting treatment with conventional anti-TB medications including; rifampicin, isoniazid, pyrazinamide, ethambutol and pyridoxine.

He was soon re-admitted following progression of his symptoms with the patient reporting side effects from the medications including blurry vision, nausea and orange urine. The patient stopped taking his medications due to these side effects. Ophthalmology review noted no objective optic neuropathy or any significant visual deficit. Re-discussion at the TB-MDT advised to momentarily hold the medications until further review. A repeat CT-T demonstrated interval reduction in the consolidative component of the left upper lobe infective change, but an increase in the size of the internal cavity measuring 7.6 cm × 2.3 cm × 2.6 cm (Fig. 2). In-vitro susceptibility testing identified sensitivity to clarithromycin/erythromycin, amikacin and linezolid with increased dose sensitivity for moxifloxacin. Following further discussion in the TB-MDT, the patient was started on clarithromycin, isoniazid, rifampicin and pyridoxine, with ethambutol avoided due to previous visual difficulties. An intensive phase of at least 3 drugs for 2 months was advised, with the final duration to be decided based on response (at least 6 months but may be 12 months total).

The patient was discharged on this amended anti-microbial regime. However, he did not attend his 3 month clinic review nor his follow up repeat CT to determine response to treatment. At the 4 month telephone clinic review, it was ascertained that the patient had significantly deteriorated and became bedbound secondary to extensive respiratory and cardiac disease. The patient reported once again he had stopped taking his medications as he was poorly tolerant of it. Due to this, he was discharged from the respiratory clinic with palliative input sought. He subsequently passed away shortly thereafter.

## Discussion

The rarity of MS as the causative organism of NTM-PD has led to limitations in the literature regarding its epidemiology, risk factors, clinical and radiological findings, diagnosis and treatment outcomes. Moore et al. conducted a review of the number of reports and rates of NTM in the UK from 1995–2006. Out of a total of 11 039 NTM reports over this time period, only 30 cases identified MS as the causative organism [4]. This indicates a prevalence rate of 0.27% of all NTM cases in the UK. NTM-PD is more common in patients with pre-existing chronic lung pathologies including COPD, asthma, cystic fibrosis and bronchiectasis. Additional risk factors include immunodeficiency and immunosuppression. MS is amongst the most pathogenic NTM species which cause NTM-PD, with cultures identifying MS almost always having a pathological significance [5, 6].

The British Thoracic Society (BTS) released guidelines for the management of NTM-PD in 2017, which provides the standards for clinical practice in the UK. Diagnosing NTM-PD requires a combination of clinical, radiographical and microbiological criteria. Criterion include the presence of respiratory symptoms, nodular or cavitary opacities on chest radiograph or multifocal bronchiectasis with small nodules on CT. Microbiological criteria include positive sputum cultures from two samples or positive culture results from one bronchial wash/lavage or lung biopsy with mycobacterial histopathological features and positive culture result [7]. The treatment recommendations presented in the BTS guidelines does not specifically cover MS NTM-PD, instead providing recommendations on more prevalent NTM species. Extrapolating BTS guidelines and recommendations from case reports would suggest concurrent treatment of rifampicin, ethambutol and isoniazid or a macrolide (clarithromycin or azithromycin). Treatment duration is advised for a minimum of 12 months [7, 8].

A case report by Croix and Munsiff discusses the progression of a cavitating lung lesion secondary to MS over a 3 year period [9]. In their case report, the male patient developed symptoms including; weight loss, productive cough, dyspnoea and fatigue. He also had a significant smoking history with a diagnosis of COPD. The chest CT showed the progressive increase in size of a large cavity in the right lung apex. Cultures obtained by video-assisted thoracoscopic surgery (VATS) grew MS. Anti-microbial therapy of rifampicin, ethambutol and azithromycin was continued for a total of 18 months, with stable CT findings at time of discontinuation.

In comparison to our case report, we can extrapolate specific findings which could be pathognomic of MS lung infections. Firstly, both male patients presented with symptoms of; productive cough, dyspnoea, weight loss and fatigue. In addition, there was the background of a significant smoking history with COPD, an underlying risk factor. Radiological similarities included the presence of an apical cavitating lesion, which demonstrated progression over time. Positive cultures were obtained by sputum in our report and VATS in theirs. In their case report, a prolonged period of anti-microbial therapy for 18 months following culture conversion yielded a successful outcome. We can extrapolate that treatment with anti-TB medications including rifampicin and ethambutol with the addition of a macrolide is potentially a sufficient treatment regime. There is evidence in the literature which suggests high levels of isoniazid resistance in-vitro amongst MS isolates [10]. Although our cultures did not specifically identify isoniazid resistance, this was considered during the course of treatment and should be factored on an individual basis. Anti-microbial therapy is advised for a 12 month period post culture conversion for other species of NTM, evidently patient specific circumstances may require a longer period of time.

Treatment response was inconclusive in our case report due to patient specific developments. The BTS guidelines advise that treatment should continue for 12 months post culture conversion for other species of NTM. Our patient had sporadic sputum culture results, with a negative repeat sputum AFB taken 2 days following the sample which ultimately identified MS. Repeat negative cultures were also taken following re-admission 11 weeks later. Although this could indicate culture conversion was obtained, but also potentially a limitation in sputum sampling or analysis given the immediate negative culture result. Due to our patients subsequent morbidity and mortality, with reduced attendance to outpatient follow up and repeat CT imaging, determining the clinical and radiological response to treatment was inconclusive. In all, the patient was treated with targeted anti-microbial medications for approximately 5 months. However, the patient experienced side effects and compliance to treatment was uncertain. This has limited the clinical effectiveness of the treatment which we can observe.

MS is a rare organism with high pathogenicity, typically infecting individuals with either weakened immune systems or underlying respiratory disease. Clinicians should be aware of such an organism as a potential cause of an atypical respiratory infection. A combination of clinical, radiological and microbiological parameters should be used in conjunction with an MDT discussion in order to guide the management of MS NTM-PD. Clinicians should remain cautious of the side effects associated with conventional anti-TB medications, with patient’s being counselled and made vigilant for any untoward effects. As an extended course of treatment is recommended, patients require monitoring and follow up assessment to ensure disease regression and resolution.

## Gurantor

Dr Aroon Sohail (corresponding author).
